# Pre-frontal Cortex Oxygenation Changes During Aerobic Exercise in Elite Athletes Experiencing Sport-Related Concussion

**DOI:** 10.3389/fnhum.2020.00035

**Published:** 2020-02-12

**Authors:** J. Patrick Neary, Carolynn M. Dudé, Jyotpal Singh, Trevor K. Len, Yagesh N. Bhambhani

**Affiliations:** ^1^Faculty of Kinesiology and Health Studies, University of Regina, Regina, SK, Canada; ^2^Independent Researcher, Moncton, NB, Canada; ^3^Faculty of Rehabilitation Medicine, University of Alberta, Edmonton, AB, Canada

**Keywords:** concussion, exercise testing, near-infrared spectroscopy, pre-frontal cortex oxygenation, physiology, athletes

## Abstract

**Aims**: Recent research suggests that aerobic exercise can be performed safely within the first week following a concussion injury and that early initiation of exercise may speed recovery. To better understand the physiological changes during a concussion, we tested the hypothesis that mild-to-intense exercise testing can be performed within days immediately following injury, and can be used to discern differences between the concussed and normal healthy state. Thus, the purpose was to observe the cerebral hemodynamic responses to incremental exercise testing performed acutely post-concussion in high-performance athletes.

**Methods**: This study was a within- and between-experimental design, with seven male university ice hockey teams participating. A subgroup of five players acted as control subjects (CON) and was tested at the same time as the 14 concussed (mTBI) players on Day 2, 4, and 7 post-concussion. A 5-min resting baseline and 5-min exercise bouts of mild (EX1), moderate (EX2), and high (EX3) intensity exercise were performed on a cycle ergometer. Near-infrared spectroscopy was used to monitor pre-frontal cortex oxy-haemoglobin (HbO_2_), deoxy-haemoglobin (HHb), and total blood volume (tHb) changes.

**Results**: ANOVA compared differences between testing days and groups, and although large percentage changes in HbO_2_ (20–30%), HHb (30–40%), and tHb (30–40%) were recorded, no significant (*p* ≤ 0.05) differences in cerebral hemodynamics occurred between mTBI vs. CON during aerobic exercise testing on any day post-injury. Furthermore, there was a linear relationship between exercise intensity vs. cerebral hemodynamics during testing for each day (*r*^2^ = 0.83–0.99).

**Conclusion**: These results demonstrate two novel findings: (1) mild-to-intense aerobic exercise testing can be performed safely as early as Day 2 post-concussion injury in a controlled laboratory environment; and (2) evidence-based objective measures such as cerebral hemodynamics can easily be collected using near-infrared spectroscopy (NIRS) to monitor physiological changes during the first-week post-injury. This research has important implications for monitoring physiological recovery post-injury and establishing new rehabilitation guidelines.

## Introduction

During the past 5–10 years, there has been a growing interest in sport-related concussion due to recent deaths in high-performance athletes (Kelly, 2001; O’Connor et al., 2017). The Centers for Disease Control and Prevention, National Center for Injury Prevention and Control have reported that there is an estimated between 1.7 and 3.8 million concussions per year in the USA alone (Rutland-Brown et al., 2006; Daneshvar et al., 2011), with approximately 30% being sport-related (Cassidy et al., 2004; Langlois et al., 2006; Gaw and Zonfrillo, 2016). Globally, it is estimated that 64–74 million individuals experience traumatic brain injury annually (Dewan et al., 2018).

Recently there has been a focus on the management of the athlete post-concussion, including when it is safe for the athlete to return-to-learn and return-to-play so that the likelihood of re-injury is decreased (Aubry et al., 2002; Collins et al., 2002; McCrory et al., 2005, 2009, 2013, 2017; Ellis et al., 2016; Terwilliger et al., 2016). Traditionally, “prescribed rest” has been used to ensure the safety of the athlete (Moser et al., 2012; Elbin et al., 2014; Thomas et al., 2015; Buckley et al., 2016). Although recent consensus statements recommend that concussed athletes must participate in a graduated, medically supervised exercise protocol before returning-to-play (Aubry et al., 2002; McCrory et al., 2005, 2009, 2013, 2017), less information is available on whether aerobic exercise can be performed immediately post-injury, i.e., with the first 72 h. However, mounting research is recommending that mild-to-moderate aerobic exercise can be performed safely within the days and weeks following injury (Leddy et al., 2016a, 2017, 2018; Bishop et al., 2017b; Dech et al., 2019), although most studies initiate exercise as a rehabilitation strategy after ~21 days post-injury (Dematteo et al., 2015; Gagnon et al., 2016; Morissette et al., 2019).

Most recently, exertional exercise testing protocols, such as the Buffalo Concussion Treadmill and Bike Test have proven to be safe and efficacious (Leddy et al., 2016a, 2018; Haider et al., 2019; Morissette et al., 2019) in evaluating the athletes post-concussion. These laboratory testing protocols have been designed with the intent to differentiate the type of concussion injury (i.e., physiologic, vestibulo-occular, cervicogenic; Ellis et al., 2014, 2015; Leddy et al., 2016a), and to discern the physiological mechanisms (cerebral autoregulation, cerebrovascular reactivity, neurovascular coupling; Ellis et al., 2015, 2016, 2018; Neary et al., 2019). This research has added to our understanding of the pathophysiological changes that occur following a concussion (Len et al., 2011, 2013; Giza and Hovda, 2014; Bishop and Neary, 2017; Bishop et al., 2017b; Worts et al., 2019), and has also provided a greater understanding of how we can apply exercise safely as a treatment strategy. Thus, it appears that evidence supports the therapeutic benefits of mild-to-moderate intensity physical exercise (Kozlowski, 2014; Hinds et al., 2016; Leddy et al., 2016a,b, 2018) and that prolonged rest may be detrimental during acute recovery post-concussion (Elbin et al., 2014; Thomas et al., 2015; Buckley et al., 2016; Wells et al., 2016).

This evolving research that has examined the pathophysiology of concussion, includes monitoring cerebral blood flow velocity using transcranial Doppler (Len et al., 2011; Wright et al., 2018), cerebral blood-oxygen-level-dependent (BOLD) changes using functional magnetic resonance imaging (fMRI; Mutch et al., 2014, 2018), blood pressure (La Fountaine et al., 2016, 2019; Bishop et al., 2017b; Wright et al., 2018), heart rate variability (HRV; Gall et al., 2004; La Fountaine et al., 2009; Bishop et al., 2017a, b), cortical activation (Pearce et al., 2019), and cerebral hemodynamics using near-infrared spectroscopy (Urban et al., 2015; Bishop and Neary, 2017). In particular, near-infrared spectroscopy (NIRS) is an optical technique that has been validated to show quantitative changes in blood volume (an indirect measure of blood flow velocity) and oxygenation in cerebral tissue (Ferrari et al., 2004; Bhambhani et al., 2006). This measurement is taken at the level of capillaries, arterioles and venules in cerebral tissue (Belardinelli et al., 1995). By measuring the concentration of both the oxygenated (HbO_2_) and deoxygenated haemoglobin (HHb), changes in total blood volume (i.e., total haemoglobin; tHb = HHb + HbO_2_) can be determined and has been suggested as a surrogate for cerebral blood flow (Wolf et al., 2007).

NIRS has been used to monitor cerebral hemodynamics in patients following concussion (Kontos et al., 2014; Urban et al., 2015; Bishop and Neary, 2017), and TBI (Bhambhani et al., 2006). The neurocognitive research by Kontos et al. (2014) and Urban et al. (2015) suggests clinical implications of using pre-frontal cortex NIRS to assess brain cognition and connectivity. Furthermore, the pre-frontal cortex has been suggested as a major area to measure these hemodynamic changes during a hypercapnic challenge (Bishop and Neary, 2017), but no studies to our knowledge are available that used NIRS during incremental exercise when assessing acute concussion within 24–48 h following injury. Collectively, these studies suggest that the vascular involvement of the pre-frontal cortex is important during an acute concussion.

Because there is mounting research focused on both physiologic changes and the importance of exercise post-concussion (Worts et al., 2019), the focus of our research was to monitor the pathophysiological changes in athletes prospectively in the days following sport-related concussion in response to acute incremental aerobic exercise. It was hypothesised that exercise could be used safely to assess the physiological status of the concussed athlete and that there would be differences in the cerebral hemodynamic response between healthy control vs. mTBI subjects. This study used an evidence-based approach to discern differences between groups to contribute to our understanding and insight into the physiological response to exercise post-mTBI.

## Methodology

### Participants and Research Ethics

Players from seven university men’s ice hockey teams in the Atlantic University Sport of the Canadian University Sport association served as potential participants for this study. All procedures were approved by the University Research Ethics Board. Before the beginning of the varsity ice hockey season, demographic information, pre-participation medical information and history, concussion history, the Physical Activity Readiness Questionnaire (PAR-Q), and written informed consent was collected from 120 (70.2%) of a total 171 eligible players. A total of 14 players sustained a diagnosed concussion, and follow-up testing was performed on the injured players. The concussed (mTBI) athlete was assessed within 48 h of the injury (D2) and then prospectively on Day 4 or 5 (D4) and Day 6–9 (D7). Logistically, it was difficult to test exactly on the same days for all athletes because of their personal schedules, so the concussed athlete was tested on a day as close to the above-mentioned days as possible. Specifically, players were tested on the following days: all subjects for **D2** were tested on day 2; for **D4**, three subjects were tested on day 5, and six subjects were tested on day 4; for **D7**, 2 subjects were tested on day 6, one subjects tested day 7, three subjects tested on day 8, and three subject tested on day 9. Five control (CON) players (one player at each university testing location where concussions occurred) were matched line-mates that exercised at the same time intervals as the concussed player. CON (matching line-mate) players were tested on the same day in an uninjured state, to alleviate any discrepancy in time of testing later in the season to control for changes in fitness level throughout the season. Table 1 shows the demographic information for each group.

**Table 1:** Summary of descriptive data for Control (*N* = 5) and Concussed subjects (*N* = 14).

Variable	Control	Concussed
*N*	5	14
Age (years)	25.0 ± 1.0	22.80 ± 1.2
Height (m)	1.79 ± 0.1	1.85 ± 0.1
Body mass (kg)	81.68 ± 8.3	90.20 ± 6.1
BMI (kg/m^2^)	25.56 ± 1.2	26.47 ± 1.2

*Values are mean ± standard deviation*.

For consistency, the medical personnel was required to use the consensus guidelines (McCrory et al., 2005) to detect and recognise a concussion. Clearance by the team physician or Certified Athletic Trainer or physical therapist was required for return-to-play.

### Procedures and Study Design

The same testing protocol (graded aerobic exercise protocol) was used for both the mTBI and CON athletes to assess pre-frontal cortex blood volume and oxygenation changes. Following orientation and verbal description of the testing protocol, a Polar S810 heart rate monitor and optical probes of the NIRS instrument were attached to the athlete. The same trained research assistant administered all testing for consistency of measurement. Once all equipment was connected, 5 min of resting baseline heart rate and pre-frontal cortex oxygenation parameters were taken simultaneously. To account for circadian rhythms, each post-concussion testing day for each mTBI athlete was scheduled at the same time of the day.

#### Exercise Testing

The exercise test lasted between 17 and 20 min and was performed on the same calibrated Monark 828E cycle ergometer (Monark Exercise, Vansbro, Sweden) throughout the study. The athlete began cycling at 40 Watts (pedaling frequency between 80–90 rpm) for a 2-min warm-up. Following the warm-up, a symptom-limited stepwise incremental exercise protocol began with each stage being 5 min in duration. The workload was increased to elicit heart rates which were mild (110–120 bpm, EX_1_), moderate (135–145 bpm, EX_2_) and intense (170–180 bpm, EX_3_), respectively. If the athlete felt they had to stop cycling, the protocol was terminated as recommended by current consensus guidelines (McCrory et al., 2005, 2009; Leddy et al., 2012). Control athletes completed an identical protocol on the same days as the injured athlete. The research assistant recorded the power output (Watts) during the first session for each athlete and this workload was repeated for all subsequent days.

#### Near-Infrared Spectroscopy

Using a spatially resolved oximeter (NIRO-300, Hamamatsu Photonics, Japan), quantitative changes in relative NIRS parameters (μM) of HbO_2_, HHb, and tHb, were measured bilaterally over the frontal lobes. The NIRS unit consists of two channels for monitoring two separate regions of tissue simultaneously. Each channel has an emitting sensor probe that generates NIR light by four laser diodes *via* an optical fibre, and two silicone diodes contained within the detector probe that absorbs NIR light between the range of 760 nm and 950 nm. The separation distance between the sensor and detector was 5 cm, which allowed the light to penetrate to a depth of approximately 2.5 cm (Ferrari et al., 2004). The probes were placed bilaterally and equidistant from the midline, approximately 1 cm above the supraorbital ridge to avoid contamination of the signal by the temporalis muscle (Kleinschmidt et al., 1996; Obrig et al., 1996). The NIRS absorbency signal was averaged over 5-s intervals. The difference between peak values during the resting baseline values and the values during the test were calculated and used for analysis (Obrig et al., 1996). The reproducibility of NIRS has been documented (Mehagnoul-Schipper et al., 2002; Koike et al., 2004; Giacalone et al., 2017), and NIRS has been used previously in concussion research (Kontos et al., 2014; Urban et al., 2015; Bishop and Neary, 2017). A Polar S810 HR monitor (Polar Electro, Kempele, Finland) was used to record heart rate during the testing protocol.

### Data Analysis

#### Data Processing

**Figure 1:**
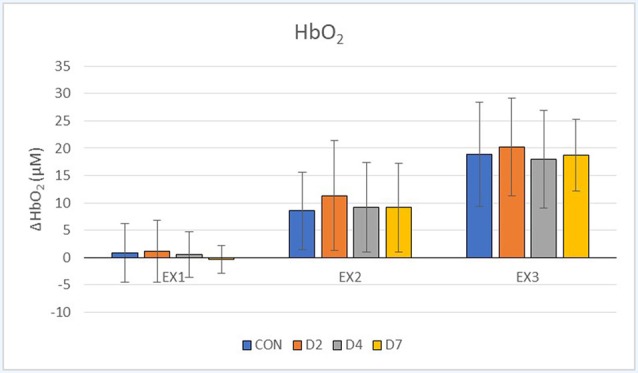
Oxyhaemoglobin (HbO_2_, μM) response during exercise testing in the CON vs. mTBI group at Day 2, 4 and 7 post-injury. EX_1_ = mild exercise (110–120 bpm), EX_2_ = moderate exercise (135–145 bpm), EX_3_ = intense exercise (170–180 bpm). Values are Mean ± Standard Deviation.

**Figure 2:**
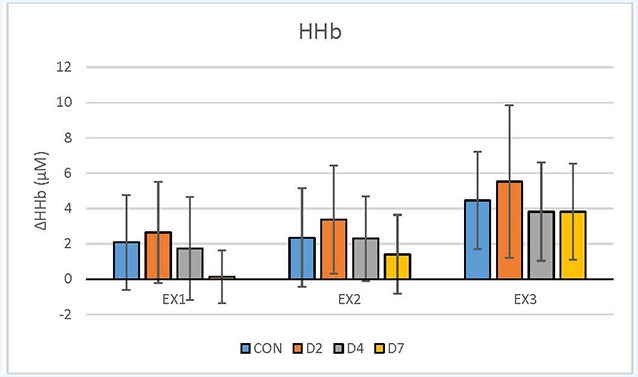
De-oxyhaemoglobin (HHb, μM) response during exercise testing in the CON vs. mTBI group at Day 2, 4 and 7 post-injury. EX_1_ = mild exercise (110–120 bpm), EX_2_ = moderate exercise (135–145 bpm), EX_3_ = intense exercise (170–180 bpm). Values are Mean ± Standard Deviation.

**Figure 3:**
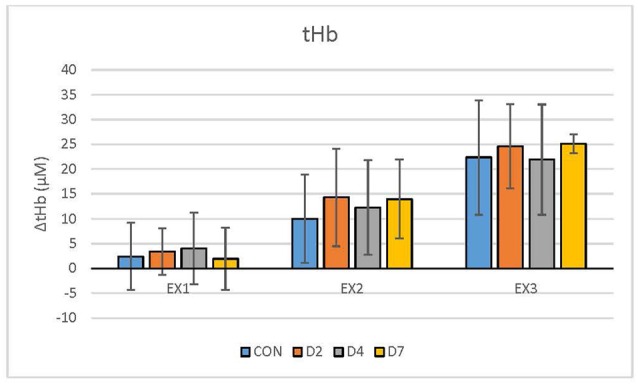
Total haemoglobin (tHb, μM) response during exercise testing in the CON vs. mTBI group at Day 2, 4 and 7 post-injury. EX_1_ = mild exercise (110–120 bpm), EX_2_ = moderate exercise (135–145 bpm), EX_3_ = intense exercise (170–180 bpm). Values are Mean ± Standard Deviation.

For descriptive purposes, the data were expressed as mean and standard deviation. All data were exported from the NIRO acquisition software at 6 Hz, and processed in Microsoft Excel (Microsoft Office Home, Redmond, WA, USA). The acquisition software was equipped with tools to convert optical density signals to relative concentration units (micromolar; μM) for oxyhaemoglobin (HbO_2_), deoxyhaemoglobin (HHb) and total haemoglobin (tHb). The average and standard deviation of the entire 5-min exercise segments are presented in Table 2 and Figures 1–3. Recovery exercise data was not included in the analysis of plots because of missing data.

**Table 2:** The NIRS variables (HbO_2_, HHb, tHb; μM) for each group, exercise condition and day of testing.

	GROUP	EX1	EX2	EX3
HbO_2_ (μM)	CON	0.85 ± 5.32	8.54 ± 7.08	18.88 ± 9.59
	D2	1.15 ± 5.60	11.36 ± 10.02	20.24 ± 8.99
	D4	0.52 ± 4.14	9.14 ± 8.18	17.96 ± 8.90
	D7	−0.36 ± 2.56	9.14 ± 8.14	18.77 ± 6.54
HHb (μM)	CON	2.09 ± 2.68	2.35 ± 2.79	4.46 ± 2.75
	D2	2.64 ± 2.87	3.37 ± 3.05	5.53 ± 4.31
	D4	1.74 ± 2.93	2.31 ± 2.40	3.83 ± 2.78
	D7	0.15 ± 1.49	1.40 ± 2.24	3.82 ± 2.72
tHb (μM)	CON	2.42 ± 6.81	9.98 ± 8.84	22.36 ± 11.54
	D2	3.40 ± 4.68	14.27 ± 9.79	24.60 ± 8.51
	D4	4.04 ± 7.19	12.26 ± 9.49	21.92 ± 11.10
	D7	1.96 ± 6.26	13.97 ± 7.93	25.12 ± 1.90

*CON, control group; D2, day 2 post-injury; D4, day 4 post-injury; D7, day 7 post-injury. EX1, mild exercise; EX2, moderate exercise; EX3, intense exercise. Values are Mean ± Standard Deviation*.

At Day 2 post-injury, one participant did not perform the third exercise segment. This resulted in five control participants completing the protocol (*n* = 4 data sets for HHb due to signal contamination), six participants completing the protocol at Day 2 post-injury (with 1 participant not completing the third exercise load; *n* = 5 data sets for tHb due to signal contamination), nine participants completing the protocol at day 4 post-injury (*n* = 6 for HHb and *n* = 8 for tHb due to signal contamination), and 7 participants completing the protocol at Day 7 post-injury (*n* = 4 for HHb due to signal contamination).

### Statistical Analyses

All NIRS data (HbO_2_, HHb and tHb) were assessed independently to observe changes between groups and conditions. One-way ANOVA’s were used to determine that differences occurred between each condition [mild exercise (EX_1_), moderate exercise (EX_2_), intense exercise (EX_3_)] between controls and participants assessed the days post-concussion. Significance was set at *p* ≤ 0.05. Due to some missing data points, an expectation maximation model with 25 iterations was used through SPSS (IBM Corp. Released 2019. IBM SPSS Statistics for Windows, Version 26.0. Armonk, NY, USA; IBM Corporation). Little’s missing completely at random test (Little, 1988) was used to ensure that the data was indeed missing at random (with significance set at *p* ≤ 0.05).

## Results

### Descriptive Data

The descriptive demographic data for the participants are presented in Table 1 and showed no differences between groups. Table 2 shows the NIRS variables for each group, exercise condition and day of testing. There were no statistically significant differences (*p* < 0.05) between the CON vs. mTBI group for any of the testing days post-injury (D2, D4, D7) for any of the oxygenation and hemodynamic (NIRS) variables (Table 2). There was a significant relationship between exercise intensity (EX_1_, EX_2_, EX_3_) vs. the NIRS cerebral hemodynamic variables during exercise (*r*^2^ = 0.83–0.99) for all days of testing.

## Discussion

We demonstrated two important findings in this study: (1) that acute, short duration mild-to-intense exercise testing (HR = 110 bpm–170 bpm) can be performed safely in the days (D2, D4, D7) immediately following the diagnosis of concussion; and (2) that physiological measures can be easily collected and can be used to add to our understanding of the pathophysiological effects of concussion. This has important implications for treatment strategies, as current research suggests that sport-related concussion or mTBI is a physiological brain injury (McCrory et al., 2017; Leddy et al., 2018), and that concussion testing can potentially differentiate the type of mTBI (Ellis et al., 2015). Furthermore, our data support recent reports that physical exercise can be used safely when assessing recently concussed athletes within the first week post-injury in a controlled laboratory environment (Leddy et al., 2016a, 2017, 2018; Haider et al., 2019).

### Basis of Physiological Concussion Testing

The primary objective of concussion testing is to characterise the physiologic response of the injured athlete. The effects of moderate-to-high intensity exercise on physiological function in the concussed state are unknown (Worts et al., 2019), and therefore we explored whether such exercise intensity could be safely maintained for a short duration (increments of 5-min workloads). To our knowledge this is the first study to systematically evaluate the physiological effects of repeated exercise tests using mild-to-intense exercise intensity (HR~ 110–170 bpm) performed within 2-days (D2) after the medical diagnosis of sport-related concussion in high-performance athletes. Based on the growing body of literature in this area, moderate-to-intense exercise testing can be performed in concussed athletes safely, but most studies were performed more than a week after injury (Baker et al., 2012; Ellis et al., 2015; Hinds et al., 2016; Leddy et al., 2016b, 2018; Haider et al., 2019; Morissette et al., 2019). Using an evidence-based approach, similar to our study, Leddy et al. (2011, 2017) developed the Buffalo Concussion Treadmill and Bike Tests to specifically evaluate symptom limitations in post-concussion syndrome (PCS) patients and showed that concussed athletes assessed on average of 33 weeks (6–36 weeks) post-injury could reliably differentiate physiological dysfunction and quantify exercise capacity in PCS patients. This test has now been used to assess the acute effects of acute concussion (Hinds et al., 2016; Haider et al., 2019). Furthermore, Ellis et al. (2015) conducted a review of the literature to describe and differentiate the type of concussion and suggested that concussed individuals can exercise to maximal volitional fatigue during supervised graded exercise testing. They classified the type of concussion as either physiologic, vestibulo-ocular, or cervicogenic (Ellis et al., 2015). It is well accepted that exercise increases the metabolic demand and stresses the autonomic nervous system function. Thus, dynamic cerebral autoregulation (Bailey et al., 2013), neurovascular coupling (Wright et al., 2017; Sharma et al., in press), heart rate (Haider et al., 2019) and HRV (Gall et al., 2004; La Fountaine et al., 2009), functional connectivity and brain activation (Kontos et al., 2014; Urban et al., 2015; Pearce et al., 2019) and blood pressure (La Fountaine et al., 2016, 2019) can be assessed to characterise the autonomic/physiological effects of acute mTBI. Therefore, this increasing body of literature demonstrates that objective physiologically based concussion testing using exercise is an important part of athlete management post-injury.

### Comparison of NIRS Hemodynamics Between Control vs. mTBI Participants

Our results showed that athletes who sustained a sport-related concussion or mTBI can undertake an incremental exercise test in 5-min stages from mild-to-intense exercise intensity on D2, D4 and D7 post-injury. Although we hypothesised that there would be significant differences between the CON and mTBI groups, the statistical analysis revealed no significant differences in the physiological responses in HbO_2_, HHb and tHb using NIRS, between groups or testing days (Figures 1–3). Previous research, including our own (Bishop and Neary, 2017), has shown that significant differences exist between days post-injury. For example, under resting conditions, HbO_2_ was statistically different (*p* ≤ 0.05) between days of recovery in mTBI participants; i.e., Day 1–3 vs. D7–14 and D4–6 vs. D7–14 (Bishop and Neary, 2017). As well, there were significant differences in the SD values between days post-injury, with the mTBI group showing reduced SD. However, it is important to note that this was in response to a hypercapnic (breath-hold) challenge which is a different paradigm than our exercise protocol. Hypercapnia has demonstrated that cerebral blood flow velocity is altered leading to cerebrovascular reactivity changes and differences between healthy controls and mTBI (Len et al., 2011, 2013; Mutch et al., 2014). Based on these observations, we extended our hypercapnic protocol (Neary et al., 2019) in the current study to include incremental (5 min) exercise workloads to assess the physiological consequences of mTBI in these athletes accustomed to intense physical exercise. No statistical differences in our hemodynamic data suggest that cerebral blood flow and oxygenation variables were not different during the exercise test in the days following acute concussion. When transitioning from rest to exercise, to sustain exercise intensity, energy (ATP) production is needed and was maintained adequately following concussion and did not compromise our athletes’ ability to complete the testing protocol.

However, a word of caution is warranted, as our data showed that differences as much as 40% (*p* > 0.05) exist between groups. Closer observation of our hemodynamic results showed that there were 30–40% differences in the mean value of tHb and HHb variables, and 20–30% differences in mean HbO_2_ between the CON vs. D2 and D4 at particular exercise workloads (EX_1_, EX_2_; Figures 1–3). However, the standard deviations in our data were extremely large (2–5 times the mean) and eta values were low (0.01–0.02 for HbO_2_, 0.06–0.12 for HHb, 0.02–0.04 for tHb) making it difficult to reach statistical significance. Based on these statistical parameters, we have estimated that approximately 45 participants per group (CON, D2, D4 and D7) are required to demonstrate statistical differences. If the participants had baseline data collected first, a within-subject design based on this effect size will require 29 participants per group to demonstrate significance. This demonstrates the importance of including baseline testing in sport-related concussion research which has been documented previously (Len et al., 2013; Neary et al., 2019).

The high SD’s are most likely related to two factors, the continuous wave NIRS device used in this study gives a relative change value and therefore differences between individual participants is common, and the small number of CON (*n* = 5) and mTBI (*n* = 14) participants in the study limited our statistical power. Individual differences could potentially nullify any differences that exist between groups as there is more variability to the data.

### Limitations of the Study

As suggested above, the major limitation of our study was the small number of participants, especially in the CON group. Future studies of this nature must increase the number of control participants, both for within- and between-group comparisons. Because of the limited number of participants in this study, the standard deviation of the oxygenation variables were quite large. This is an inherent limitation of continuous-wave NIRS technology in that it gives a relative change value in oxygenation concentration (μM). Typically, the best method to deal with relative differences between participants is to conduct a resting baseline and then zero the relative changes from the resting baseline (Bishop and Neary, 2017). We did this in our study to account for differences between participants but still, the SD values were high. Based on the SD and eta values we estimated that 45 subjects would be needed to show statistical significance, or based on a within-subject design, 29 participants per group. Other limitations included not collecting rating of perceived exertion or symptom scores (Sport Concussion Assessment Tool; SCAT) to see how each athlete felt after their test, and whether this changed from D2 to D7. Anecdotally, the players reported feeling that exercise on D2 was difficult for them but felt better by D7 post-injury. However, all athletes were able to complete all the testing days. Recent research by Morissette et al. (2019) showed that symptomatic subjects when assessed at day 45 post-injury reported significantly higher ratings of perceived exertion vs. controls, although metabolic demands were not different at the same workload. The inclusion of a symptom checklist before and after the exercise testing may have provided additional information about their rating of perceived exertion, and considered for future research. However, the reader is reminded that SCAT scores are subjective and cannot always be relied upon. Finally, ECG recordings instead of using an HR monitor would have allowed us to calculate HRV changes from day-to-day. Previous research has demonstrated that HRV is altered in acute concussion (Gall et al., 2004; Papaioannou et al., 2008). Based on our results, and because of these limitations, it was not possible to determine the extent to which autonomic dysfunction occurred in our mTBI participants (Bishop et al., 2017b), or to characterise the type of concussion (Ellis et al., 2015).

## Conclusion

This research demonstrated that a mild-to-intense incremental exercise test can be used safely within 2 days of injury, and can be repeated during the initial 7–9 days to assess brain physiology. This is the first study to demonstrate this finding in highly trained athletes with acute brain injury. Furthermore, regardless of whether statistical differences were apparent between groups or days of testing in this study, our results provide value physiological information on the status of the frontal lobe of the brain during the acute phase of sport-related concussion.

### Future Directions and Implications

We believe the future direction in concussion research is to continue to characterise the injured athlete physiologically and clinically following mTBI, and use the results to develop a physiologically based exercise prescription and treatment strategy that can be individualised to the athlete. Although we did not find statistically significant differences between the CON vs. mTBI group on days of testing, the physiological/clinical significance of the acute changes in the NIRS measurements indicates that concussed athletes are able to exercise intensely for short durations (5 min) in the days following a concussion (days 2–7). These results suggest that the brain is still able to generate motor output to the muscles and cardiovascular system, despite having altered autonomic function (Len et al., 2011, 2013; Mutch et al., 2014). The practical implication of this is that clinicians can recommend short duration exercise as a viable alternative to try to enhance recovery, albeit, at mild-to-moderate intensity exercise appears more beneficial. Research by Haider et al. (2019) supports this contention and demonstrated that recently concussed (<10 days) individuals could exercise safely at subthreshold heart rates. Furthermore, based on our results, it is our recommendation that mild-to-intense physical exercise testing must become part of the assessment protocol in contact sports where there is an increased risk and incidence for mTBI to occur, and testing of this nature must be included in future consensus guidelines.

## Data Availability Statement

The datasets for this study can be found by contacting the corresponding author.

## Ethics Statement

The studies involving human participants were reviewed and approved by University of New Brunswick Research Ethics Committee. The patients/participants provided their written informed consent to participate in this study.

## Author Contributions

Inception and design of the study were conceived by CD and JN. CD collected and analysed all data. JS and TL contributed to specific sections and assisted in the statistical analyses. All authors contributed to the writing and editing of the article.

## Conflict of Interest

The authors declare that the research was conducted in the absence of any commercial or financial relationships that could be construed as a potential conflict of interest.
